# Establishing extended pluripotent stem cells from human urine cells

**DOI:** 10.1186/s13578-023-01051-1

**Published:** 2023-05-16

**Authors:** Chunfang Hao, Shilong Chu, Xiongzhi Quan, Tiancheng Zhou, Junjie Shi, Xiaofen Huang, Guangming Wu, Micky Daniel Tortorella, Duanqing Pei

**Affiliations:** 1 CAS Key Laboratory of Regenerative Biology, Guangdong Provincial Key Laboratory of Stem Cell and Regenerative Medicine, Guangzhou Institutes of Biomedicine and Health, Chinese Academy of Sciences, Guangzhou, 510530 Guangdong China; 2 University of Chinese Academy of Sciences, Beijing, 100049 China; 3 Division of Basic Research, Guangzhou Laboratory, Guangzhou, 510005 China; 4 Laboratory of Cell Fate Control, School of Life Sciences, Westlake University, Hangzhou, 31003 China

**Keywords:** EPSC, O-IPSC, hUC-EPSCs, Differentiation

## Abstract

**Background:**

Extended pluripotent stem cells (EPSCs) can contribute to both embryonic and trophectoderm-derived extraembryonic tissues. Therefore, EPSCs have great application significance for both research and industry. However, generating EPSCs from human somatic cells remains inefficient and cumbersome.

**Results:**

In this study, we established a novel and robust EPSCs culture medium OCM175 with defined and optimized ingredients. Our OCM175 medium contains optimized concentration of L-selenium-methylcysteine as a source of selenium and ROCK inhibitors to maintain the single cell passaging ability of pluripotent stem cells. We also used Matrigel or the combination of laminin 511 and laminin 521(1:1) to bypass the requirement of feeder cells. With OCM175 medium, we successfully converted integration-free iPSCs from easily available human Urine-Derived Cells (hUC-iPSCs) into EPSCs (O-IPSCs). We showed that our O-IPSCs have the ability to form both intra- and extra- embryonic chimerism, and could contribute to the trophoblast ectoderm lineage and three germ layer cell lineages.

**Conclusions:**

In conclusion, our novel OCM175 culture medium has defined, optimized ingredients, which enables efficient generation of EPSCs in a feeder free manner. With the robust chimeric and differentiation potential, we believe that this system provides a solid basis to improve the application of EPSCs in regenerative medicine.

**Supplementary Information:**

The online version contains supplementary material available at 10.1186/s13578-023-01051-1.

## Background

Extended pluripotent stem cells (EPSCs) have been shown to be able to contribute to both embryonic and extraembryonic tissues when injected to early stage embryos [1, 2]. Therefore, EPSCs are closer to the totipotent cell than embryonic stem cells (ESCs) [3–6]. Despite progresses made in recent years, it remains necessary to optimize methods for EPSC generation. To this end, multiple chemicals, such as inhibitors or activators of signaling pathways are tested to induce EPSCs [1, 2, 7, 8]. Interestingly, signaling pathways such as WNT, MAPK, GPCR, PI3K-AKT, AMPK seem to reach dynamic equilibrium based on single cell RNA sequencing data of human early embryo-zygote and hESC [9], indicating that addition of chemicals to activate or inhibit these pathways as in the pulished protocols might not be essential for the generation of EPSCs. For hEPSCs, two reports provided evidence that they can differentiate into hepatocytes and cardiomyocytes [10, 11]. Given these recent progresses, it is desirable to establish alternative methods to generate EPSCs and better characterize their differentiation potentials both in vitro and in vivo. To this end, we report here a novel feeder-free culture system with defined ingredients to generate EPSCs capable of generating three germ layers and extraembryonic cells.

## Results

### Establishment of EPSCs with OCM175 medium

To minimize the usage of chemical compounds in conversion of PSCs to EPSCs, we re-analyzed the single cell sequencing data of human preimplantation embryo and ESC reported previously [9]. We revealed that signaling pathways such as MAPK, WNT and AMP were similar between zygotes and ESC (Additional file 1: Fig. S1). Based on this insight, we attempted to establish a medium (OCM175, Table 1) for generating EPSCs without inhibitors or activators. We formulated COM175 with equal DMEM/F12 and KO-DMEM (1:1) as basic medium (DMEM/F12 provides rich nutrients and HEPES balance system, while KO-DMEM has high glucose and osmotic pressure close to the embryonic culture medium). We included the same amount of bFGF2 as the mTeSR1 and E8 medium, but less TGFβ because high concentration of TGFβ inhibits cell proliferation, 2 μg/mL Transferrin, less VC to decrease the potential of mesoderm development, EAA to mimic the human early preimplantation embryo culture medium, L-seleno-methylselenocysteine (L-SeMC) used to provide organic selenium, and less GlutaMAX-I and Transferrin to inhibit ferroptosis. Because we found that ferroptosis were one of main cell death ways during pluripotent stem cells passage, selenium and GlutaMAX-I transferrin were included based on these result (Additional file 2: Fig. S2).

**Table 1:** OCM175 medium components (50 mL)

Component	Volume added	Storage Concentration
bFGF2	250 μL	20 μg/mL
IGF2	8 μL	0.5 mg/mL
TGFβ1	2.5 μL	10 μg/mL
Insulin	100 μL	10 mg/mL
L-Selenium-Methylselenocysteine	5 μL	100 μg/mL
Transferrin	2 μL	50 mg/mL
GSH	200 μL	50 mg/mL
VC	20 μL	70 mg/mL
NEAA	500 μL	100 ×
EAA	250 μL	200 ×
GlutaMAX-I	100 μL	500 ×
Heparin	10 μL	10 mg/mL
HSA	200 μL	100 mg/ml
B27	1 mL	50 ×
NaHCO_3_	800 μL	0.25 M
KO-DMEM	25 mL	
DMEM/F12	20.6 mL	
B27	1 mL	
Osmotic pressure (mosm/kgH_2_O)	~ 311	

**Fig. 1:**
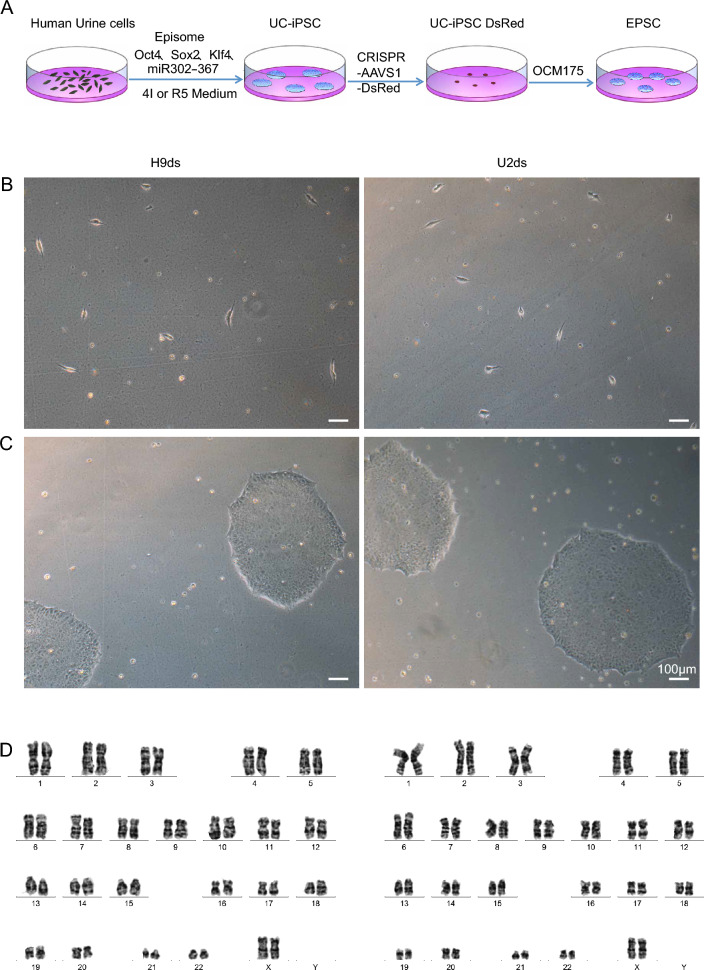
Process of the EPSC formation and the formula of EPSC medium. **A** Schematic diagram of the EPSC formation. **B** Single cell cultured in OCM175 Medium. **C** hUC-iPSCs cultured 7d in OCM175 named O-IPSC. **D** Karyotype analysis. Scale bar: 100 μm

To test the ability of our OCM175 medium in conversion of PSCs to EPSCs (Fig. 1A), we decided to establish iPSCs from Human Urine-derived cells (hUCs). We first electro-transfected episomal plasmids pCEP4-E02S-T2K and pCEP4-miR302-367 into the hUCs, and induced the transfected cells with R5 Medium as described previously [12] and the 4I medium (Additional file 3: Fig. S3; Additional file 6: Table S1). We then utilized CRISPR/Cas9 to insert the DsRed to AAVS1 after we selected the monoclonal hUC-iPSCs. Finally, we cultured the hUC-iPSCs in OCM175 medium for 6–10 days and we named the resulted cells as O-IPSC (Fig. 1B and C). Karyotype assay showed that our O-IPSCs have correct sets of chromosomes (Fig. 1D).

### Characterization of EPSCs generated in OCM175 (O-IPSC)

**Fig. 2:**
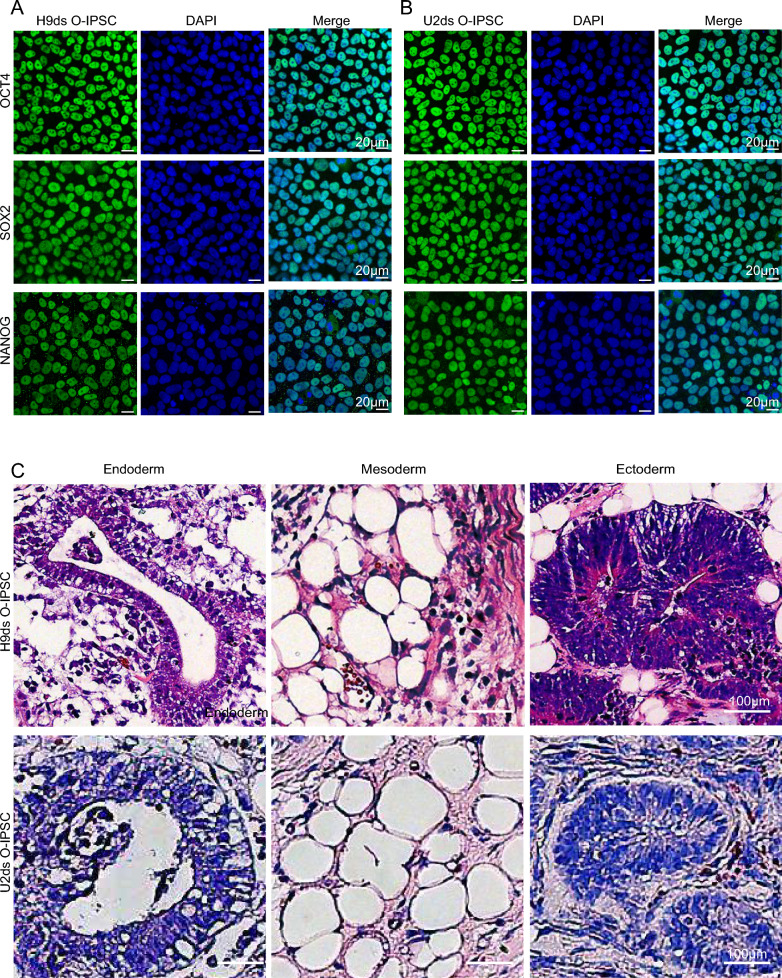
Immunofluorescence of O-IPSCs and teratoma experiments. **A**, **B** OCT4, SOX2 and NANOG were positive staining of H9ds and U2ds O-IPSCs. Scale bar: 20 μm. **C** H9ds and U2ds O-IPSCs could be differentiate to three germ layers by teratoma experiment, such as Endoderm: glandular tube; Mesoderm: adipocyte and Ectoderm: neural tube. Scale bar: 100 μm

**Fig. 3:**
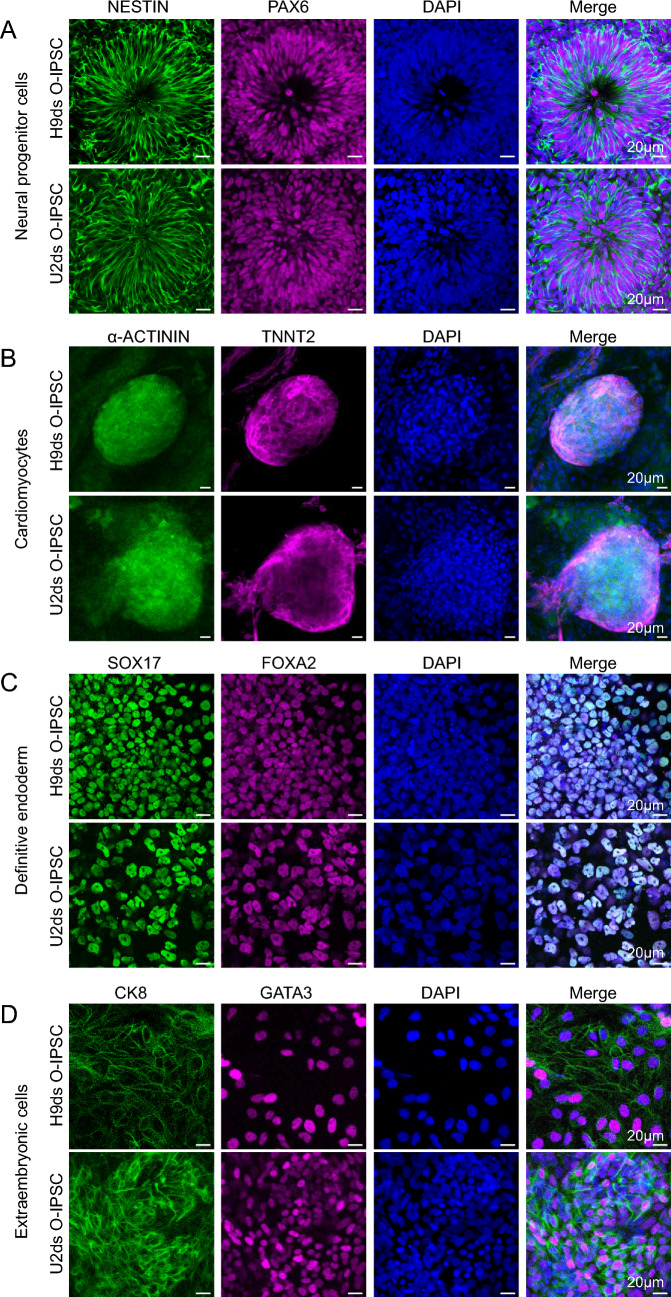
Differentiation ability of three germ layers and extraembryonic cells.** A** NESTIN and PAX6 were positive staining of neutral progenitor cells. **B** SOX17 and FOXA2 were positive staining of definitive endoderm cells. **C** α-ACTININ and TNNT2 were positive staining of cardiomyocytes. **D** CK8 and GATA3 were positive staining of extraembryonic cells. Scale bar: 20 μm.

To measure the pluripotency of O-IPSC, we monitored the expression of *OCT4, NANOG and SOX2* by immunofluorescence assay. As shown in Fig. 2A and B, our O-IPSCs expressed all three marker proteins. In addition, HE staining of teratoma showed that O-IPSC have the ability to differentiate into all three germ layers (Fig. 2C). Furthermore, O-IPSC could be differentiated to neuronal progenitor cells (NPC), definitive endoderm cells (DEC), cardiomyocytes (CM) and extraembryonic cells (Fig. 3A–D). The results indicated that O-IPSCs are pluripotent.

**Fig. 4:**
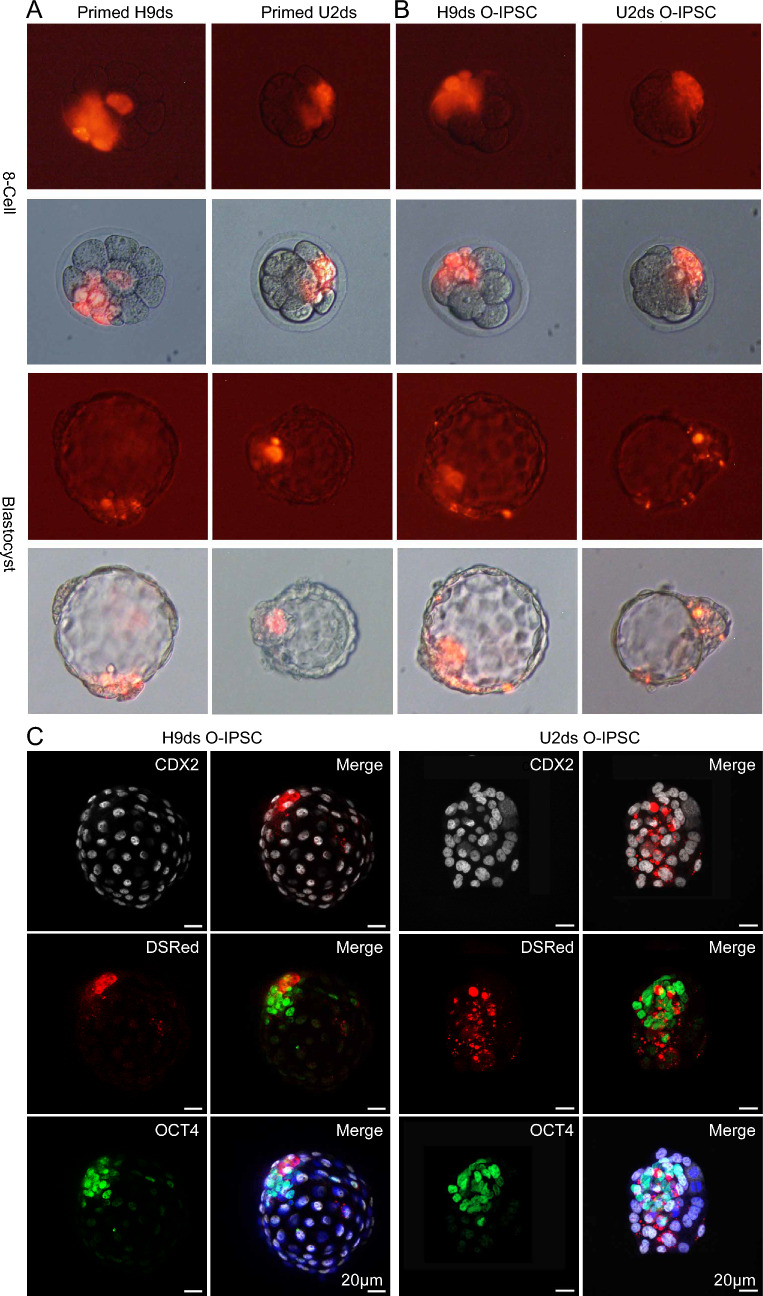
8-cell injection and chimeric blastocyst experiments. **A** H9ds and U2ds injected into 8-cell embryo and cultured to blastocyst. **B** H9ds O-IPSC and U2ds O-IPSC injected into 8-cell embryo and cultured to blastocyst. **C** H9ds O-IPSC U2ds O-IPSC were respectively co-localized with CDX2 positive trophoblast cells and OCT4 was positive inner cell mass (ICM). Scale bar: 20 μm

**Fig. 5:**
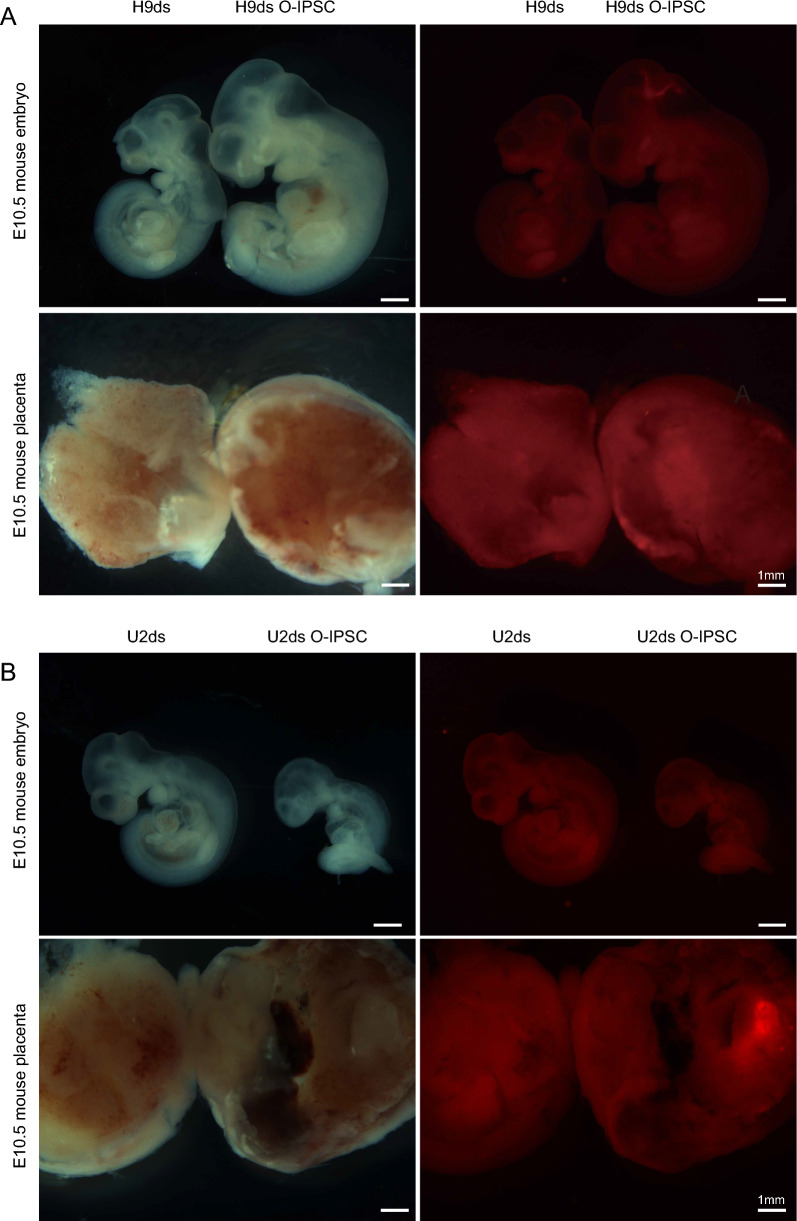
Chimeric experiment of embryo injection in vivo. The localization of cells from mTeSR1 (PSC) and OCM175 (O-IPSC) in E10.5 embryo and placenta: **A** H9ds cells (Left) and H9ds O-IPSC (Right) in E10.5 embryo and placenta. **B** U2ds (Left) and U2ds O-IPSC (Right) in E10.5 embryo and placenta. Scale bar: 1 mm

To show the O-IPSCs derived from OCM175 medium really have the characteristic of the extended pluripotent stem cells, we injected our O-IPSCs into embryo to form chimeric blastocysts in vitro. As shown in Fig. 4A and B, we could observe the O-IPSC in the ICM and TE of E3.5-E4.5 blastocyst. In order to detect the localization of O-IPSCs in the blastocyst, we stained the blastocyst with CDX2 and OCT4 antibodies. Immunofluorescence assay showed that the O-IPSCs had red fluorescent localized with the signal of *OCT4* and *CDX2*, indicated that the O-IPSCs have ability to chimeric in ICM and TE cells (Fig. 4C). More importantly, the experiment of mouse E10.5 chimeric embryos further verified that O-IPSCs had the ability of intra- and extra-embryonic chimerism (Fig. 5).

In our culture system, we used Matrigel or combination of Laminin511 and Laminin521 as culture matrix to support EPSCs cell growth and proliferation in OCM175. We found that our cells could be cultured in this system for two days without changing medium from 4–5 days, especially for later period of cell culture (Additional file 4: Fig. S4). In order to achieve the possibility of clinical application in the future, we showed that K115, a ROCK inhibitor and a drug for glaucoma and ocular hypertension could also be used to support O-IPSCs passaging as efficient as Thiazovivin (Additional file 5: Fig. S5).

## Discussion

Different from other reported EPSC protocols, our OCM-175 medium contains only one inhibitor, i.e., the ROCK inhibitor, significantly less than reported previously [13, 14]. The addition of ROCK inhibitor allows for successful passaging of EPSCs. In addition, we also showed that an alternative ROCK inhibitor K115 [15], which is an approved drug for the treatment of glaucoma and ocular hypertension [16], could maintain the passaging of O-IPSCs. Furthermore, L-SeMC, an organic selenium, which is low toxic to normal cells rather than tumor cells [17–19]. We also show that IGF2 can improve the chimerism of EPSCs based on the fact that it is an imprinted gene and its role in implantation [20–22]. Another potentially interesting finding is that ferroptosis contributes to death of pluripotent stem cells, apart from apoptosis previously reported [23–27]. Ferroptosis is a consequence of lipid peroxidation [28–32]. This finding may be further explored for deriving or culturing EPSCs in the near future.

## Methods

### Establishment of urine iPSC

Six well culture plate was coated with 0.1% gelatin for 30 min in advance. Human urine was collected in 50 mL tube and centrifuged at 1200 rpm, 10 min, in the presence of P/S (Penicillin/Streptomycin). Collected cells were plated into the pre-coated 6 well culture plate with 3 mL UC medium with 5 μg/mL Primocin, which were cultured in a CO_2_ incubator with daily medium change until reach 70% cell confluence for transfection. To obtain urine iPSC: on Day 1, the plasmids (6 μg pEP4 EO2S ET2K and 4 μg pCEP4-miR302-367) were electrotransferred into the UC cells and cultured in REGM urine cells culture medium (Lonza, cc-4127) in 10 cm culture plate pre-coated with Matrigel (Corning, 35427); on Day 2, cells were passaged to 2–4 wells of Matrigel pre-coated 6 well culture plate with 1:1 REGM medium: 10% FBS DMEM mixture; on Day 3, 4I or 5R medium were added and medium was changed every two days. About 8 days in R5 medium or 12 days in 4I medium, mTesR1 (STEMCELL, 05850) medium was then used according to the shape of cells till the pluripotent stem cell clone formed.

### Establishment of DsRed monoclonal iPSC using CRISPR/Cas9

200 × 10^4^ hUC-iPSCs were transfected with 3 μg Puc57-DsRed-Neo plasmid or Puc57-AAVS1-EF1a-DsRed-PA-NEO and 2 μg CRISPR-CAS9 AAVS1-DsRed plasmids by electrotransfection. Cells were seeded into the 6 well culture plate pre-coated by Matrigel in 3 mL mTeSR1 with 1.2 μM Thiazovivin (Selleck, S1459). After 24 h, the medium was changed to mTeSR1 with 120 μg/mL G418 for 2–3 day. The DsRed cells were sorted by flow cytometry (MoFlo Astrisos) to 96 well culture plate and 6 well culture plate to obtain the monoclonal pluripotent stem cells.

### The establishment of EPSC

Plate was pre-coated with 1:100 Matrigel diluted with Knock out-DMEM (gibco, 10829018) at 37 ℃ for 2 h-3 h or overnight. Alternatively, mixture of 0.5 μg/mL iMatrix-511 (Nippi, 892011) and 10 μg/mL laminin 521 (BioLamina, LN521-05) in PBS with Ca^2+^ and Mg^2+^ were used to coat 12 well plate at 37 °C for 2 h or overnight. Pluripotent stem cells were detached to single cell by Accutase (STEMCELL, 07920) for 10 min. 8 × 10^3^–2 × 10^4^ cells were seeded to pre-coated 12 well culture plate and cultured in OCM175 medium for 6–10 days. From day 0 to day 1, cells were cultured with 1.2 μM Thiazovivin in OCM175 medium. On Day 2 and 3, the medium was changed to OCM175. From day 4–5 culturing medium was changed every day or every 2 days.

### Immunofluorescence

Cells grown on slides were fixed by 4% PFA for 30 min, permeated by PBS with 0.2% Triton-X 100 for 30 min, and blocked 1 h by 3% BSA. Indicated primary antibodies: OCT4 (Santa Cruz, sc-5279), NANOG (CST, 4903) and SOX2 (R&D, MAB2018) were added for 2 h in R.T. or 4 °C overnight. Secondary antibody (Alexa Fluor 488 goat anti-mouse IgG, Invitrogen, A11001; Alexa Fluor 488 goat anti-rabbit IgG, Invitrogen, A11008) 1:200–1:500 was added for 1 h at RT. Cells were mounted with antifade mounting solution (Vector, H-1000) after 2.5 μg/mL DAPI (Sigma-Aldrich, D9542) for 1 min. Washed in PBS three times between each step. Images were taken with the Zeiss 710 NLO confocal microscope.

### Immunofluorescence of embryos

The embryos were fixed by 4% PFA for 30 min in 96 well plate and washed with 0.1% PVA in PBS for 3 times. Embryos were then permeated with 0.2% Triton-X 100 and blocked with 3% BSA. 10 μL of 1:100 diluted primary antibody OCT4 and CDX2 (Cell Signaling Technology, 3977) was added to the embryos and then covered in mineral oil in 3.5 cm or 6 cm culture plate for 4 °C overnight. Secondary antibody (Alexa Fluor 488 goat anti-mouse IgG, Invitrogen, A11001; Alexa Fluor 633 goat anti-rabbit IgG, Invitrogen, A21070)  was used at 1:200 dilution for 1 h. The embryos were washed in PBS three times between each step. Finally the embryos were mounted with antifade mounting solution after  2.5μg/mL DAPI (Sigma-Aldrich, D9542) for  3min, and images were taken with  the Zeiss 710 NLO confocal microscope.

### Karyotype analysis

iPSCs and EPSCs were cultured in 3 mL mTeSR1 and OCM175 culture medium followed by treatment with 100 μL colcemid (20 μg/mL) (DAHUIBIO) for 1 h. G-banding of chromosomes in metaphase was then performed. Cells were collected and 8 mL 0.075 M KCl preheated at 37 °C were added with vigorous shaking mix. Cells were then incubated at 37 °C for 25 min, span down and fixed in 2 mL fixative solution (methanol: glacial acetic acid = 3:1) for 10 min, after which supernatant was discarded. After 3 washes with  8 mL fixative solution, the cells were suspended in the fixative solution with suitable cell concentration, and dripped from a certain height to a slide precool at 4 °C. The slides was dried at 85 °C for 3 h, digested with trypsin for 20 s–30 s and stained with Giemsa (gibco, 10,092–013) to obtain visual chromosomes. At least 20 metaphases were analyzed per cells. Karyotyping was described according to the International System for Human Cytogenetic Nomenclature (ISCN).

### Teratoma differentiation and extra-embryonic hematoxylin–eosin staining assay

Single cell of pluripotent stem cell cultured in OCM175 medium were resuspended in 200 μL of medium and co-injected subcutaneously with 200 μL Matrigel in the axilla of immune deficiency B-NDG mice (Biocytogen). Teratomas generally developed within 3 to 6 weeks. The injected mice were sacrificed before tumor size exceeded 20 mm in diameter and the teratomas were isolated and fixed in 4% PFA. After sectioning, teratomas were analyzed by hematoxylin–eosin staining assay. All animal experiments were performed in compliance with protocol # 1031–088-16 from the Committee on Animal Care at GIBH.

### 8C-injection and E10.5 chimera mice experiment

8-cell embryos of ICR mouse (Beijing Vital River Laboratory Animal Technology) were collected  according to our protocol, then 10 single cells cultured in OCM175 were injected into the 8-cell embryos by microinjection. After injection, the embryos were cultured in KSOM with 1.2 μM Thiazovivin for 4 h, and then cultured in KSOM for 24 hours, removed zona pellucida by Acidic Telluride solution, then cultured to E4.5 stage in an incubator with 5% CO_2_. These embryos were examined by immunofluorescence to detect whether the chimeric cells in ICM and TE cells.

Meanwhile, other injected 8cell embryos were  cultured in KSOM with 1.2 μM Thiazovivin for 4 h and then transferred to KSOM till for transplanting into 2.5dpc pseudopregnant mice and the E10.5 mouse embryos were collected  to evaluate the chimerism  by the LEICA Fluorescence stereomicroscope. 

### Three germ layer differentiation

NPC inductionwas carried out according to the published protocol [33]. The culture plates were precoated with Matrigel instead of laminin. Taking notice that cell density must be 100 percent to start induction.  The tic-tac-toe style mass was made by pipette tip to passage from well to another well on day 8.

 For cardiomyocyte induction, 8 μM CHIR-99021 (Selleck, S1263) instead of 12 μM CHIR-99021, was added during the induction [34, 35].

The progenitor of mesoderm induction was done according to the protocol of Dr. TC Pan et al. [36].

### Induction of extraembryonic cells

5 × 10^4^ O-IPSC cells were seeded to 24 well plate pre-coated with Matrigel in TSCM2 medium with 1.2 μM Thiazovivin for 2 days. TSCM3 medium was renewed every day from day 3 to day 6. TSCM2 medium consists of: 1 μM PD0325901 (Selleck, S1036), 5 μM A83-01 (Selleck, S7692), 250 μM LPA (Tocris, 325465-93-8), 0.8 mM VPA (Selleck, S3944), 20 ng/mL FGF4 (PEPROTECH, 100-31), 70 μg/mL VC (Sigma-Aldrich, V900134), 2% ITS (gibco, 41400045), 100 μg/mL QsrHSA (Oryzogen, HYC002M01), 32.7 μM Ethanolamine (Selleck, S6210), GlutaMAX-I (100× , gibco, 35050-061), Sodium pyruvate (100× , gibco, 11360-070). TSCM3  was made of TSCM2 medium with addition of 5% KSR and without VPA.

### Flow cytometry

Cells were detached by Accutase to single cell and resuspended by mTeSR1 or 0.4% BSA in PBS. After 1 wash with PBS, cells were centrifuged at 180*g* for 5 min and resuspended to 100 × 10^4^/mL. Filter cells were analyzed by flow cytometry (MoFlo Astrisos).

### Measurement of osmic pressure in culture medium

Osmic pressure of culture medium was measured according to the instruction of manufacture (OM806 osmometer, YASN).

### Orbitrap of dead cells and analysis of ferroptosis

The dead cells were collected and used for protein expression analysis by Orbitrap Fusion Lumous (Thermo Fisher) as described by Zhang XF et al. [37], then data were analyzed by R language.

## Supplementary Information

**Additional file 1: Figure S1.** The signaling pathway were reanalyzed between human zygote and hESC. The signaling pathway were reanalyzed between Zygote and ESC P10 from Tang’s single cell RNA-SEQ data. Red color: up-regulation of signaling pathway, Blue color: down-regulation of signaling pathway, fold change > 2, p < 0.05.

**Additional file 2: Figure S2.** The major cell death pathways of pluripotent stem cell and orbitrap protein analysis. **A** Pluripotent stem cells after passage for 24 h. **B** Amplified cell death picture. **C** Heatmap of orbitrap analysis: from Left to Right: H9, supernatant death cells of H9S, hUC-iPSC-2, supernatant death cells of U2S. **D** The major cell death ways of pluripotent stem cells. **E** Venn diagram of up-regulation and down-regulation of protein expression in H9S U2S contrast with H9 and U2.

**Additional file 3: Figure S3.** The process of integration-free iPSCs induction from human urine-derived cells. **A** The process of human urine cells was inducted to be iPSCs. pEP4 EO2S ET2Kand pCEP4-miR302-367were electroporated into human urine cells, and the process of cell shape changing. **B** The monoclonal hUC-iPSCs by 4I induction medium and karyotype analysis. **C** The monoclonal hUC-iPSCs by R5 induction medium and karyotype analysis. Scale bar: 100 μm.

**Additional file 4: Figure S4.** DsRed cells in mTeSR1 and OCM175 culture medium and different culture conditions. **A** H9ds and U2ds O-IPSCs were cultured in mTeSR1. **B** H9ds and U2ds O-IPSCs were cultured in OCM175. **C** H9ds and U2ds O-IPSCs were cultured in mTeSR1 and OCM175 without changing medium for 2 days.

**Additional file 5: Figure S5.** Clinical drug K115 could be used as a Rock inhibitor for subculture of pluripotent stem cells. **A** Day 1 of H9, hUC-iPSC-2, hUC-iPSC-3 passaging by mTeSR1 with Thiazovivin. **B** Day 4 of H9, hUC-iPSC-2, hUC-iPSC-3 passaging. **C** Karyotype analysis of H9, hUC-iPSC-2, hUC-iPSC-3 or the 10th generation by Thiazovivin. **D** Day 1 of H9, hUC-iPSC-2, hUC-iPSC-3 passaging by mTeSR1 with K115. E Day 4 of H9, hUC-iPSC-2, hUC-iPSC-3 passaging. F Karyotype analysis of H9, hUC-iPSC-2, hUC-iPSC-3 for the 10th generation by K115. Scale bar: 100 μm.

**Additional file 6: Table S1.** Composition of 4I induction Medium” has been changed to” Composition of 4I induction Medium for hUC-iPSCs.

## Data Availability

Availability of data and materials The datasets and materials presented in the current study are available from the corresponding author on request.

## Abbreviations

EPSC: Extended pluripotent stem cell
hUC-EPSCs: Extended pluripotent stem cells from human urine-derived cells
PSC: Pluripotent stem cell
iPSC: Induced pluripotent stem cell
ESC: Embryonic stem cell
hUC-iPSCs: Induced pluripotent stem cells from human urine-derived cells
O-IPSC: IPSCs cultured in OCM175
H9ds: H9 DsRed
U2ds: hUC-iPSC-2 DsRed
TGF-β: Transforming growth factor beta
bFGF2: Basic fibroblast growth factor 2
IGF2: Insulin-like growth factor 2
ROCK: Rho-associated protein kinase
INS: Insulin
VC: Vitamin C
ECM: Extracellular matrix
GSH: Glutathione
