# Supplementary figures and images for: Establishing extended pluripotent stem cells from human urine cells
Source: Cell Biosci. 2023 May 16;13:88. doi: 10.1186/s13578-023-01051-1 (PMC10186642; doi:10.1186/s13578-023-01051-1)

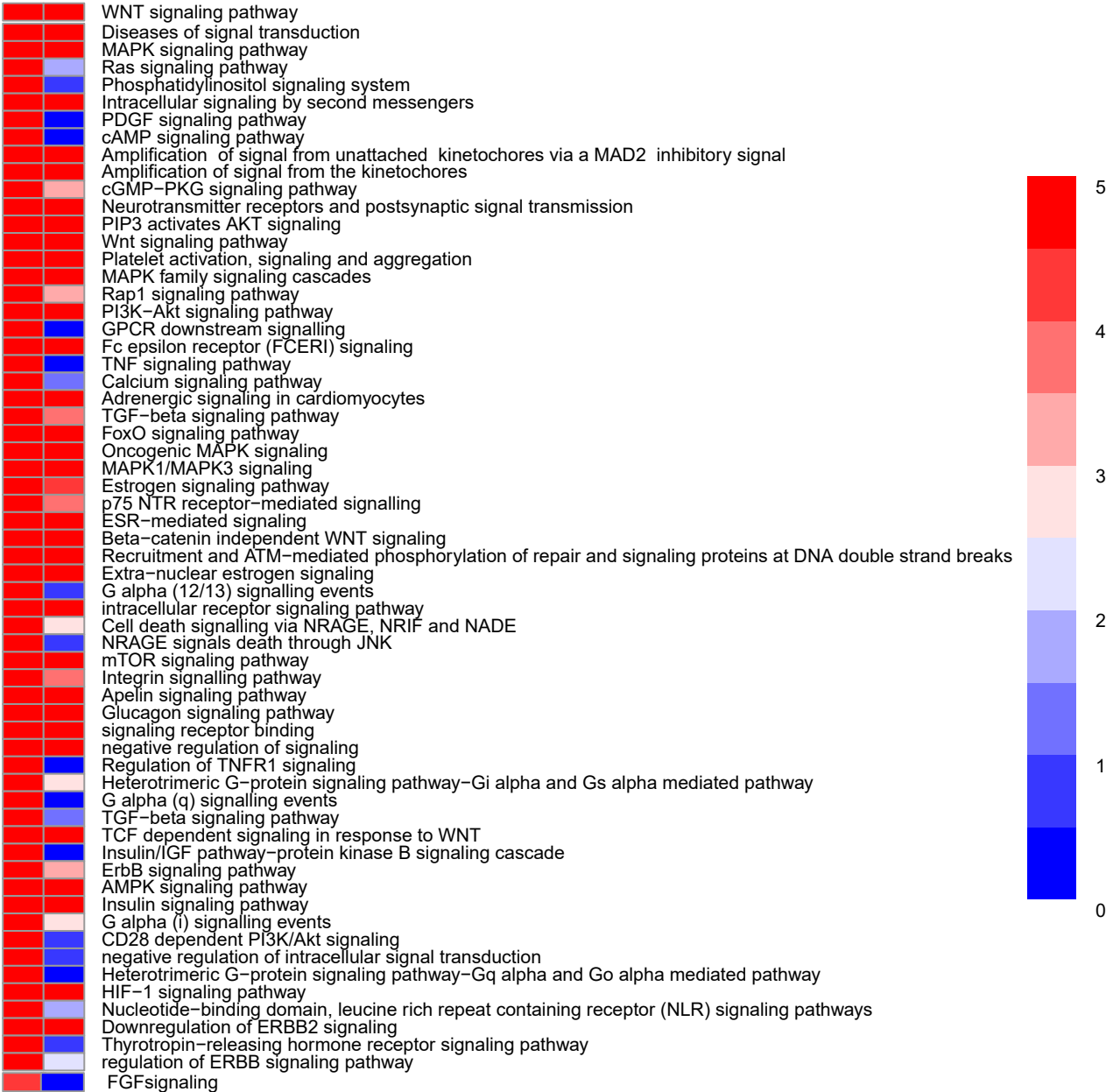

ESCP10.Zygot\_e\_up\_pvalues

ESCP10.Zygot\_e\_down\_pvalues

Supplement: Supplementary file 1 — Additional file 1: Figure S1. The signaling pathway were reanalyzed between human zygote and hESC. The signaling pathway were reanalyzed between Zygote and ESC P10 from Tang’s single cell RNA-SEQ data. Red color: up-regulation of signaling pathway, Blue color: down-regulation of signaling pathway, fold change > 2, p < 0.05. [file 13578_2023_1051_MOESM1_ESM.pdf]

A

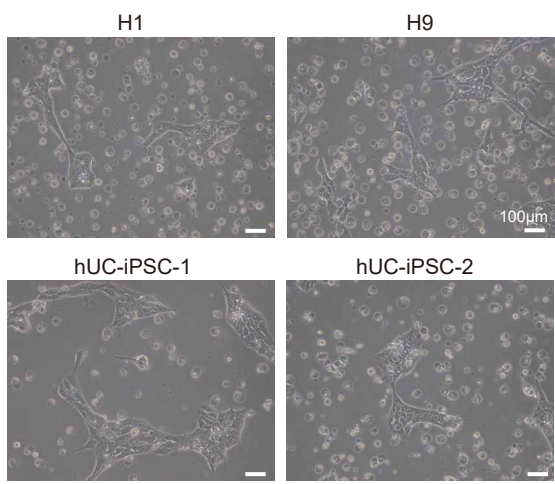

B

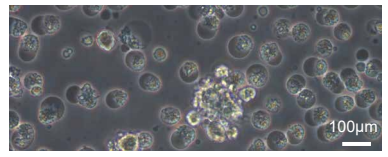

D

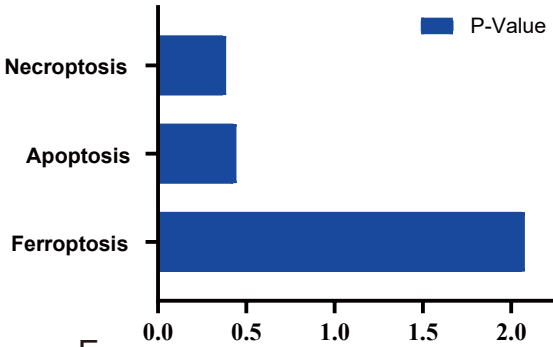

C

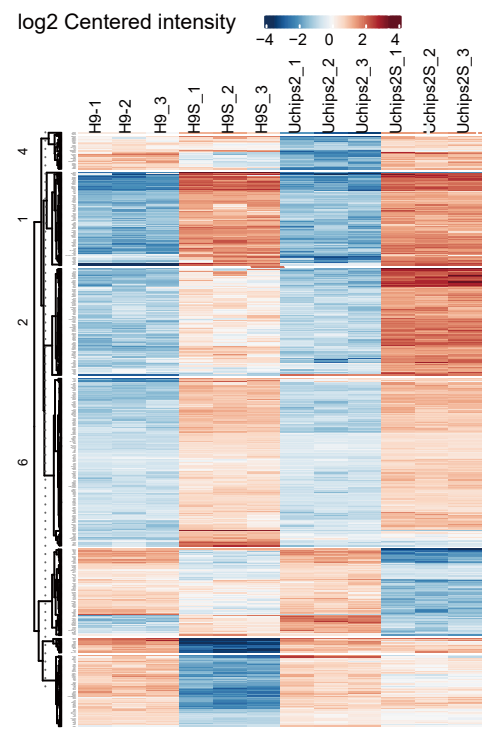

E

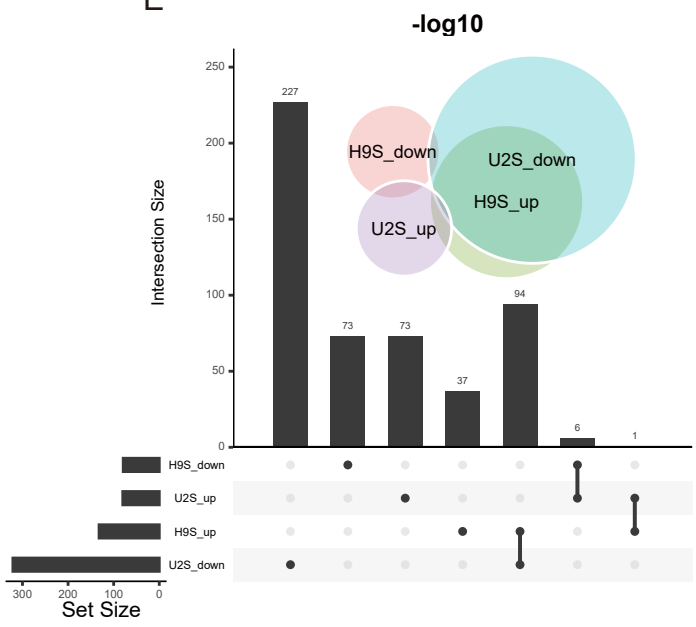

Supplement: Supplementary file 2 — Additional file 2: Figure S2. The major cell death pathways of pluripotent stem cell and orbitrap protein analysis. A Pluripotent stem cells after passage for 24 h. B Amplified cell death picture. C Heatmap of orbitrap analysis: from Left to Right: H9, supernatant death cells of H9S, hUC-iPSC-2, supernatant death cells of U2S. D The major cell death ways of pluripotent stem cells. E Venn diagram of up-regulation and down-regulation of protein expression in H9S U2S contrast with H9 and U2. [file 13578_2023_1051_MOESM2_ESM.pdf]

mTeSR1/Matrigel

OCM175/Matrigel

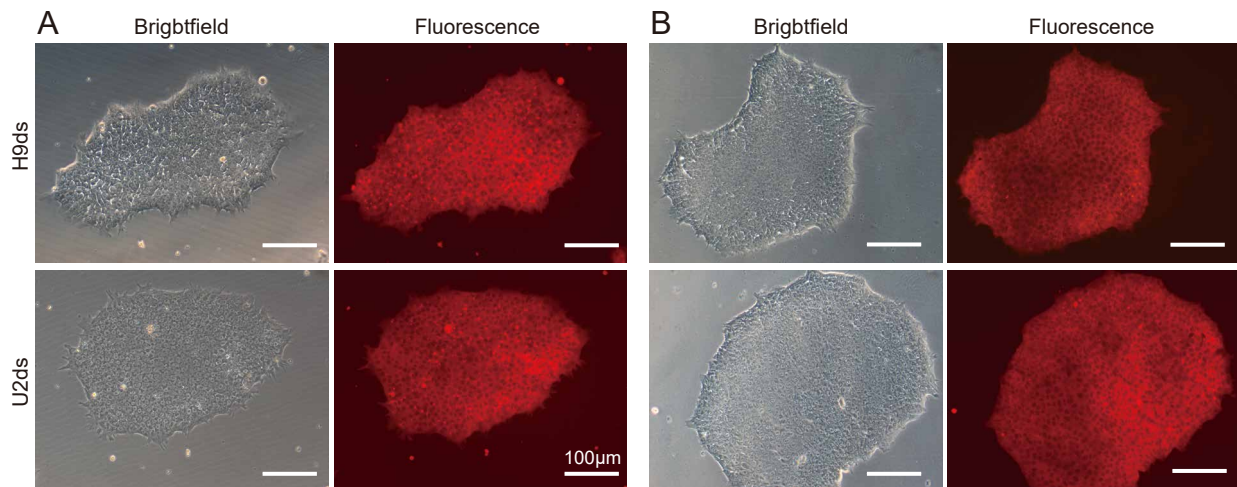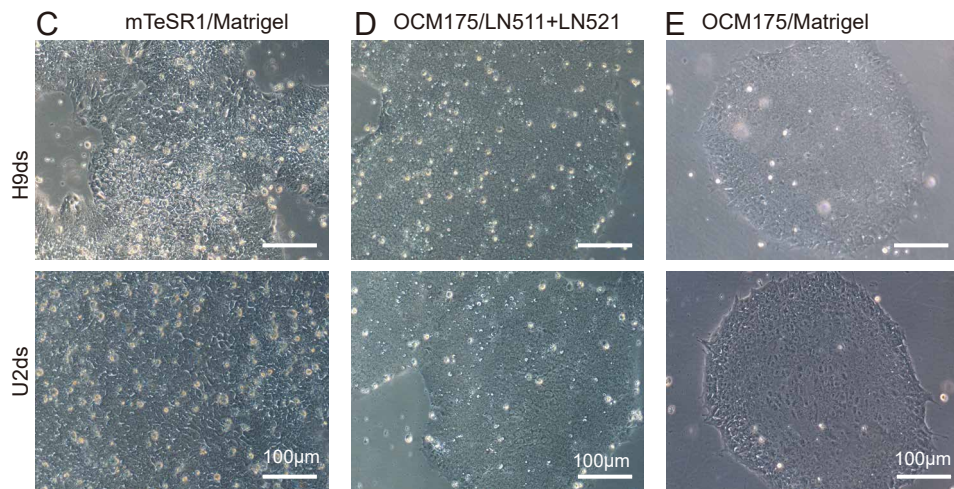

Cell growth Without changing medium for 2 or 3 days

Supplement: Supplementary file 4 — Additional file 4: Figure S4. DsRed cells in mTeSR1 and OCM175 culture medium and different culture conditions. A H9ds and U2ds O-IPSCs were cultured in mTeSR1. B H9ds and U2ds O-IPSCs were cultured in OCM175. C H9ds and U2ds O-IPSCs were cultured in mTeSR1 and OCM175 without changing medium for 2 days. [file 13578_2023_1051_MOESM4_ESM.pdf]

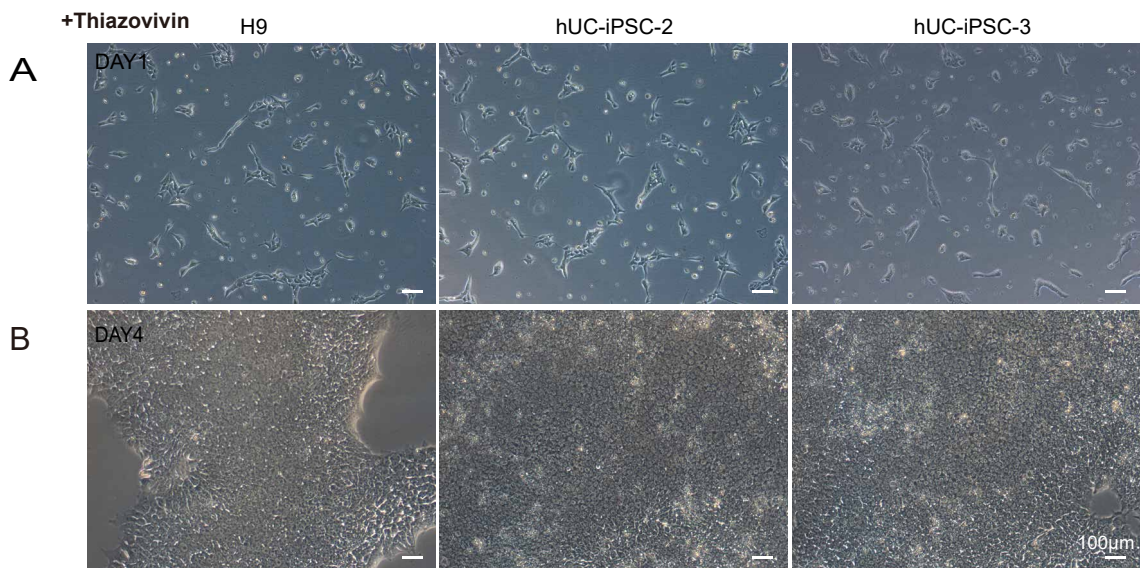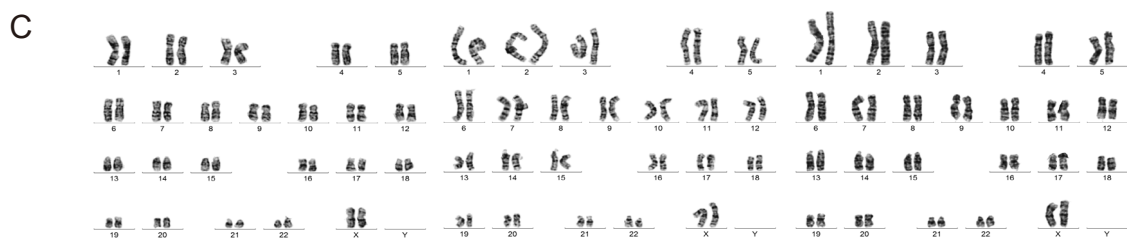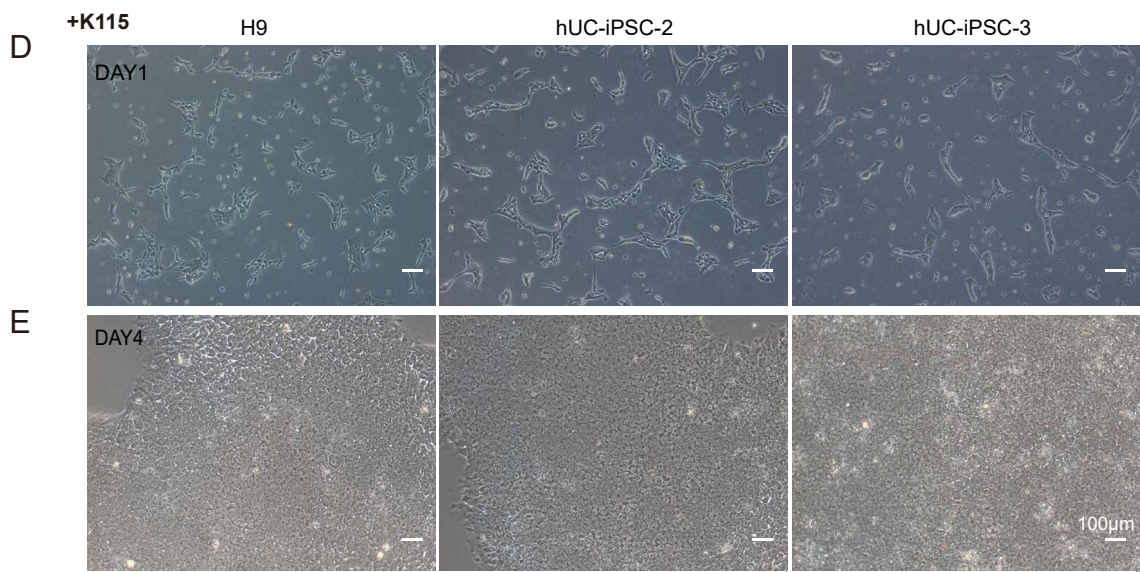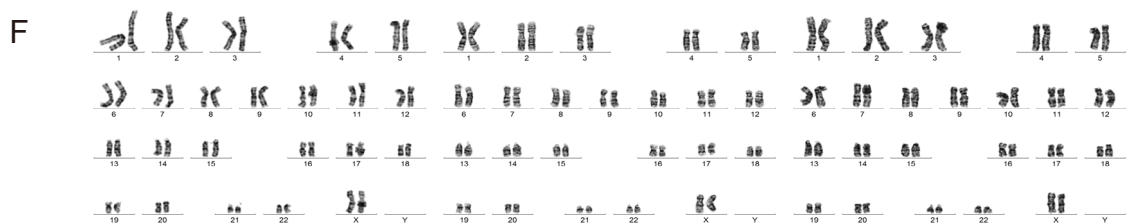

Supplement: Supplementary file 5 — Additional file 5: Figure S5. Clinical drug K115 could be used as a Rock inhibitor for subculture of pluripotent stem cells. A Day 1 of H9, hUC-iPSC-2, hUC-iPSC-3 passaging by mTeSR1 with Thiazovivin. B Day 4 of H9, hUC-iPSC-2, hUC-iPSC-3 passaging. C Karyotype analysis of H9, hUC-iPSC-2, hUC-iPSC-3 or the 10th generation by Thiazovivin. D Day 1 of H9, hUC-iPSC-2, hUC-iPSC-3 passaging by mTeSR1 with K115. E Day 4 of H9, hUC-iPSC-2, hUC-iPSC-3 passaging. F Karyotype analysis of H9, hUC-iPSC-2, hUC-iPSC-3 for the 10th generation by K115. Scale bar: 100 μm. [file 13578_2023_1051_MOESM5_ESM.pdf]
