# Supplementary material for: Establishing extended pluripotent stem cells from human urine cells
Source: Cell Biosci. 2023 May 16;13:88. doi: 10.1186/s13578-023-01051-1 (PMC10186642; doi:10.1186/s13578-023-01051-1)
Supplement: Supplementary file 3 — Additional file 3: Figure S3. The process of integration-free iPSCs induction from human urine-derived cells. A The process of human urine cells was inducted to be iPSCs. pEP4 EO2S ET2Kand pCEP4-miR302-367were electroporated into human urine cells, and the process of cell shape changing. B The monoclonal hUC-iPSCs by 4I induction medium and karyotype analysis. C The monoclonal hUC-iPSCs by R5 induction medium and karyotype analysis. Scale bar: 100 μm. [file 13578_2023_1051_MOESM3_ESM.pdf]

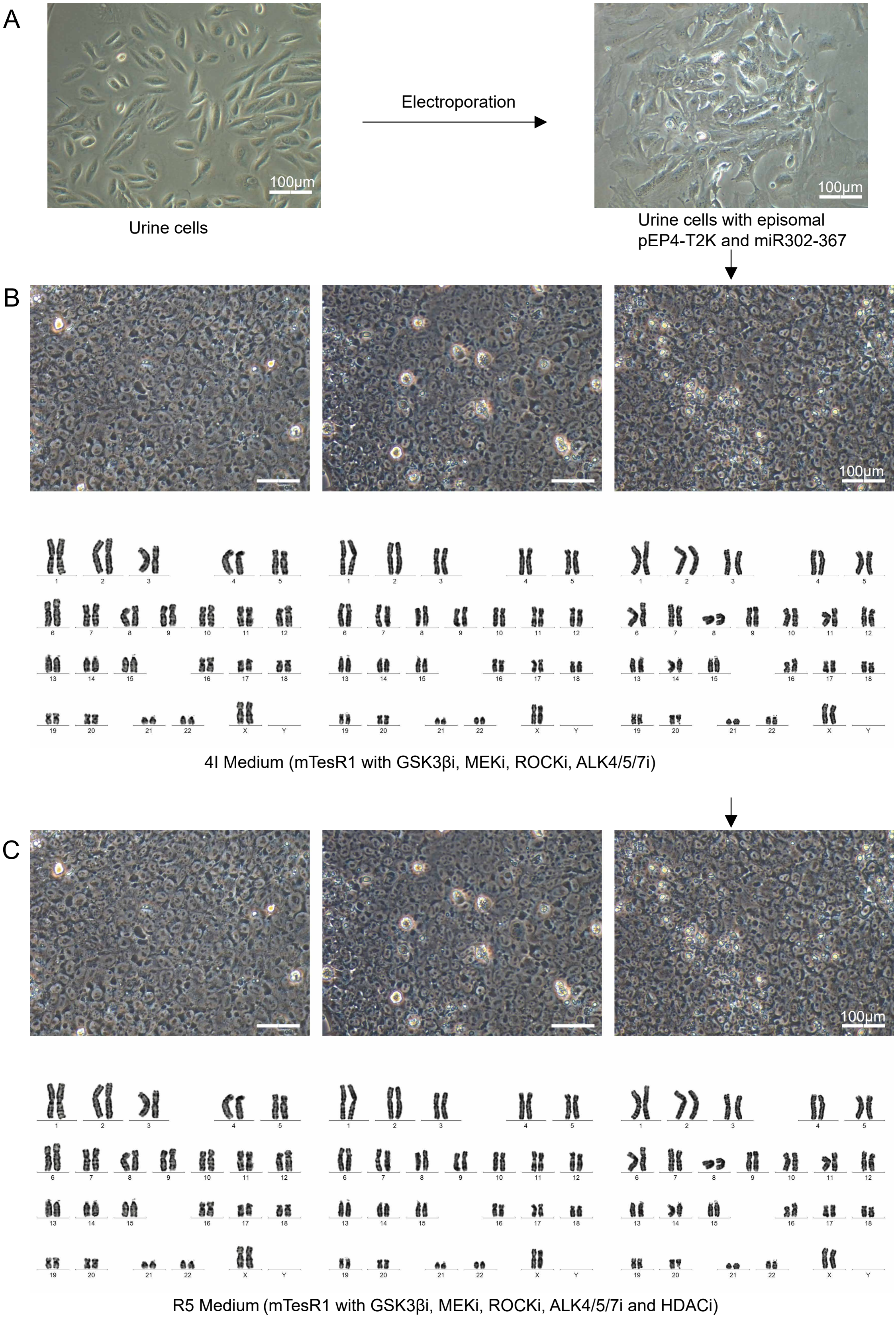
A
Electroporation
100µm
100µm
Urine cells
Urine cells with episomal
pEP4-T2K and miR302-367
B
100µm
4I Medium (mTesR1 with GSK3βi, MEKi, ROCKi, ALK4/5/7i)
C
100µm
R5 Medium (mTesR1 with GSK3βi, MEKi, ROCKi, ALK4/5/7i and HDACi)
