# Supplementary material for: Establishing extended pluripotent stem cells from human urine cells
Source: Cell Biosci. 2023 May 16;13:88. doi: 10.1186/s13578-023-01051-1 (PMC10186642; doi:10.1186/s13578-023-01051-1)
Supplement: Supplementary file 6 — Additional file 6: Table S1. Composition of 4I induction Medium” has been changed to” Composition of 4I induction Medium for hUC-iPSCs. [file 13578_2023_1051_MOESM6_ESM.pdf]

Table S1 Composition of 4I induction medium

Reagent	Working concentration
PD0325901	0.5 μM
A83-01	0.5 μM
Thiazovivin	3 μM
CHIR-99021	3 μM
mTeSR1	
